# Antitoxins in the Prevention and Treatment of Disease

**Published:** 1900-01

**Authors:** E. A. Woldert

**Affiliations:** Philadelphia


Abstracted for Texas Medical Journal.

### Antitoxins in the Prevention and Treatment of Disease.

Under the above caption this subject is treated by Surgeon J. J. Kinyoun (M. H. S.) in an issue of the *Forum.* The writer divides the subject into the results obtained as well as a historical review of the different kinds of sera, including that for diphtheria, tetanus, anti-venomous serum, sera of bubonic plague, cholera; and has, also, words to say relative to typhoid fever and smallpox.

He first calls attention to the error of confusing immunity with the results of curative treatment, pointing out that in the former instance the immunity brought about by an attenuated virus does not differ from that established by an attack of the disease; whereas in the case of curative treatment, the germs of disease are rendered nugatory, owing to the fact that the poisons generated by them are completely neutralized. Fodor and Nuttall, in the year 1884, first demonstrated what is now known as immunization. Shortly afterward (1889) Roux and Yersin discovered the poison of diphtheria, and ascertained its influence on the course of the disease, and in the same year experiments were made in the cure of the disease by the use of the blood of animals which had recovered from the infection; Babes and his colaborators experimented with the serum in rabies. In 1891 Behring and Kitasato published their result in the treatment of animals suffering from tetanus by the use of blood serum highly immunized against this disease; and shortly afterward Behring published his first paper on the serum therapy of diphtheria. The writer then speaks of the subject of the preparation of the diphtheria antitoxin, and the result attending its use in different regions of the world, showing that in each instance the number of deaths from diphtheria has, in each instance, always decreased after its administration.

Beginning with Berlin it is found that from the years 1886 to 1897 the number of deaths had not decreased up to 1894 (the year of the introduction of antitoxin), after which time the number of deaths constantly decreased. These results are quite striking, as may be observed by a glance at the mortality table. Thus in 1886 the number of admissions in the Berlin hospitals were 1738, and the number of deaths were 609; 1889, admissions 1623, number of deaths 573; 1892, number of admissions 2070, number of deaths 837; 1893, admissions 2450, number of deaths 951.

Now follows proof of the value of diphtheria antitoxin which seems more than a coincidence, and arguments along this line cannot hold good. In the year 1894 (the year of the introduction of antitoxin), the number of admissions were 2890, and the number of deaths 801. In this year from March to September all cases of *diphtheria* and *croup* admitted to the Kaiserin Friedrich Hospital in Berlin were treated by antitoxin. From September to the middle of November no serum could be obtained. During this period the mortality for all cases rose to 48.8 per cent.—the same rate as that prevailing before antitoxin had come into use. As soon, however, as a further supply of the serum was obtained, the death rate again sank to the *former figure*. Another instance occurred in the hospitals of Prague, Vienna and Trieste, which also conclusively demonstrated the efficacy of antitoxin. In these three cities no serum could be obtained for a period of six weeks, and note the death rate; thus, in Prague the death rate rose to 53 per cent.; in Vienna to 65.6 per cent.; and in Trieste to 50 per cent. During the previous six weeks in these three cities when antitoxin was used the mortality was as follows: In Prague, 12.7 per cent.; in Vienna, 18.7 per cent.; and in Trieste, 22 per cent.

In Paris, where the number of cases of diphtheria and croup are not reported, but only the number of deaths, it has been found that there has been a reduction in the number of deaths quite as marked as in these other cities; thus, in 1886 the number of deaths from these two diseases were 1524; in 1887, 1564; in 1892, 1363; in 1893, 1398; in 1894, 1262; in 1895, 993; in 1896, 411; and in 1898 the number of deaths, from both diphtheria and croup, had fallen to only *274*.

In London, in 1894, the death rate was 61.8 per cent., as compared with 17.2 per cent. for 1898, when antitoxin had become generally used.

In America the most gratifying results have been obtained. The death rate from diphtheria and croup in Boston for the years 1880-1894 was 30.75 per cent.; while for the ensuing years 1895-1897 it was only 12.61 per cent. In Chicago prior to 1895 the mortality was 30.36 per cent., and within the past year this has been reduced to less than 10 per cent.

In the treatment of croup notable results have been obtained as the following shows; thus, O'Dwyer collected reports of 5546 cases of laryngeal diphtheria and membranous croup occurring before 1892, when the death rate was 69.5 per cent.; Monti collected a series of 12,736 cases previous to 1897, and found the death rate to be 73.3 per cent. All this has now been changed as evidenced by the report of the American Pediatric Society, which states that in 1074 cases of croup the percentage of *recoveries* was 78.8 per cent., as contrasted with a previous *death rate* of 70 per cent.

The writer next considers the supposed ill effects of antitoxin, such as paralysis, heart and kidney complications, giving it as his opinion that the reason paralyses are more frequently observed than was formerly the case, that at the present time under the treatment by antitoxin more cases *recover* from the disease.

Regarding the antitoxin for tetanus the statement is made that the serum possesses immunizing properties far superior to the antitoxin for diphtheria; but its curative effect has been disappointing. Statistics are presented by Kinyoun showing that in eighteen cases in which the serum has been injected into the cerebrum twelve recoveries have ensued. Owing to its immunizing effect the serum has been employed with the most gratifying results in cases of wounds where tetanus infection has been suspected. Regarding anti-venene or anti-venomous serum prepared by Calmette, while the serum is derived from the cobra family, it is equally efficacious in neutralizing the venom of other poisonous serpents, such as the viper and rattlesnake. Statistics show that in India 20,000 persons die annually from the bite of serpents, but under the treatment by anti-venene improvement has been shown. Of six persons bitten by the cobra—almost invariably fatal—all have recovered, while one case of moccasin bite has been treated by Kinyoun with very satisfactory results. To be of benefit this remedy must be administered very soon after the bite has occurred.

Other sera have been made, such as anti-plague, anti-streptococcus, anti-cholera and anti-tubercle, but as yet such experiments have not been very successful, with the possible exception of that used in cholera.

According to the investigation of Haffkine a serum has been prepared by him which has a marked influence against the disease. Two preparations have been made, a stronger and a weaker, known as No. 1 and No. 2. These vaccines are inoculated subcutaneously, commencing with the weaker (No. 2) and following within eight or ten days with the stronger. Haffkine, in his report to the Government of India, states that from 1895-1896, 42,176 persons received anti-cholera inoculations, of which number 22,703 submitted to the first, and 19,473 to both, with the result that in a district in which an epidemic of cholera was prevalent, the disease was almost eradicated. Among 6534 inoculated cases 27 cases of cholera developed after completion of treatment and 24 deaths ensued; while in 13,226 cases not inoculated 227 cases developed, with 210 deaths. The immunity effected by anti-cholera inoculation appears to last for some time, for in 4000 persons so treated at Calcutta only four contracted the disease, and this after the lapse of more than two years.

Of the antitoxin for bubonic plague, in the opinion of the writer, the consensus of opinion seems to be that, while the anti-plague serum has the power of protecting animals inoculated with it, it exerts a curative power *only* at a very *early stage* of the disease, producing little, if any, effect if administered at a later period. The death rate does not appear to have been materially lowered by its use. An anti-plague vaccine has also been prepared by Haffkine, and in the case of 14,035 persons who have taken the treatment only 84 cases have developed; while in 8026 persons, living in the same environment and under identical conditions, who were not inoculated 2147 contracted the disease, of which 1800 ended fatally.

E. A. WOLDERT, M. D., Philadelphia.

December 30, 1899.

